# Cytokine Regulation from Human Peripheral Blood Leukocytes Cultured* In Vitro* with Silver Doped Bioactive Glasses Microparticles

**DOI:** 10.1155/2019/3210530

**Published:** 2019-06-11

**Authors:** Jefferson Muniz de Lima, Edlainne Pinheiro Ferreira, Roberta Ferreti Bonan, David Nascimento Silva-Teixeira, Luiz Ricardo Goulart, Joelma Rodrigues de Souza, Eliton Souto de Medeiros, Paulo Rogério Ferreti Bonan, Lúcio Roberto Cançado Castellano

**Affiliations:** ^1^Human Immunology Research and Education Group-GEPIH, Escola Técnica de Saúde da UFPB, Universidade Federal da Paraíba, João Pessoa, Brazil; ^2^Institute of Health Sciences, Department of Clinical Medicine, Universidade Federal do Triângulo Mineiro Federal, Uberaba, Brazil; ^3^Laboratory of Nanobiotechnology, Institute of Genetics and Biochemistry, Universidade Federal de Uberlandia, Uberlandia, Brazil; ^4^Department of Medical Microbiology and Immunology, University of California Davis, Davis, CA, USA; ^5^Department of Physiology and Pathology, Universidade Federal da Paraíba, João Pessoa, Brazil; ^6^Materials and Biosystems Laboratory, Universidade Federal da Paraíba, João Pessoa, Brazil

## Abstract

Bioactive glasses (BG) applications include tissue engineering for bone regeneration, coating for implants, and scaffolds for wound healing. BG can be conjugated to ions like silver, which might add some antimicrobial properties to this biomaterial. The immunomodulatory activity of ion-doped bioactive glasses particles was not investigated before. The aim of this work was to evaluate the cytotoxic and immunomodulatory effect of BG and silver-doped bioactive glass (BGAg) in human peripheral blood cells. BG and BGAg samples belonging to the system 58SiO_2_*•*(36-x)CaO*·*6P_2_O_5_*·*xAg_2_O, where x = 0 and 1 mol%, respectively, were synthesized via sol–gel method and characterized. Cytotoxicity, modulation of cytokine production (TNF-*α*, IL-1*β*, IL-6, IL-4, and IL-10), and oxidative stress response were investigated in human polymorphonuclear cells (PMNs) and peripheral blood mononuclear cells (PBMCs) cultures. Cell viability in the presence of BG or BGAg was concentration-dependent. In addition, BGAg presented higher PBMCs toxicity (LC50 = 0.005%) when compared to BG (LC50 = 0.106%). Interestingly, interleukin4 was produced by PBMCs in response to BG and BGAg in absence of phytohemagglutinin (PHA) and did not modulate PHA-induced cytokine levels. Subtoxic concentrations (0.031% for BG and 0.0008% for BGAg) did not change other cytokines in PBMCs nor reactive oxygen species (ROS) production by PMN. However, BG and BGAg particles decreased zymosan-induced ROS levels in PMN. Although ion incorporation increased BG cytotoxicity, the bioactive glass particles demonstrated a* in vitro* anti-inflammatory potencial. Future studies are needed to clarify the scavenger potential of the BG/BGAg particles/scaffolds as well as elucidate the effect of the anti-inflammatory potential in modulating tissue growth* in vivo*.

## 1. Introduction

Bioactive glass (BG) consists of a SiO_2_ network, having P_2_O_5_ as an adjuvant and CaO and Na2O as modifiers [1, 2]. The bioactivity of this material allows its application in the field of regeneration and tissue engineering [3]. It can be used in a wide range of applications, such as bioactive fillers in bone regeneration [4], coating for implants, dental grafting [5, 6], and scaffold for tissue repair, with porous arrangements similar to trabecular bone [3, 7]. BG is most used as hard tissue replacement material, although some studies show remarkable properties in soft tissues repair, as observed in decreased blood coagulation time, angiogenesis, and reduced wound healing time [8].

Recently, BGs have been associated with inorganic materials such as ions for nonbone therapeutic applications [9]. Silver-doped glasses showed antibacterial and antifungal effect against* Escherichia coli, Staphylococcus aureus* [10–12],* Pseudomonas aeruginosa,* and* Candida albicans *[13] in comparison to neat BG. Such proprieties may minimize complications on bone surgery like bacterial infection by topical drug delivering in a controlled and continuous manner [14]. However, silver loading may increase hypersensitivity, chronic inflammation, and immune stimulation due to materials exposure [15].

The potential immunomodulatory activity of bioactive glasses has been tested before [7, 16]. Results indicated that differences in immune response modulation are dependent on material composition or on a particular system from which the bioactive glasses are selected. Some samples indicated an ability to inhibit the secretion of inflammatory cytokines in the presence of an inflammatory stimulus [16]. However, the immunomodulatory activity of bioactive glasses doped with silver ions has not been investigated before. Little is known about the effects of Ag^2+^ on healthy primary cells of the human immune system. The complete understanding of the specific interactions and response dynamics of the immune system to different materials is still inconclusive, especially for health applications or safety recommendations [17]. Therefore, the aim of this work was to evaluate the cytotoxic and immunomodulatory effect of BG and silver-doped bioactive silica over human leukocytes.

## 2. Materials and Methods

### 2.1. BG Synthesis

Samples belonging to the system 58SiO_2_•(36-x)CaO*·*6P_2_O_5_*·*x Ag_2_O with x = 0 or 1 mol% (Neat BG and BGAg) were previously synthesized and fully characterized by physical-chemical analysis and gently provided by Pires et al. [18]. Briefly, hydrolysis and condensation of tetraethyl orthosilicate (TEOS), calcium nitrate tetrahydrate (Ca(NO_3_)_2_•4H_2_O), triethyl phosphate (TEP; Sigma Aldrich), and silver nitrate (AgNO_3_; PlatLab) were used to obtain the gels. The molar ratio of EtOH: TEOS was of 1:1. The other precursors were dissolved in distilled water. The pH of solutions was adjusted to 2 by addition of HNO_3_. The obtained gels were dried for 3 days at room temperature and 2 days in a drying oven, at 120°C. The dried BG gels were heated up to 700°C for 1/2h, at a constant rate of 3°C min^−1^. Herein, the glasses were passed through a 200-mesh British Standard Sieve (final particles diameter smaller than 74*μ*m). The samples synthesis was performed under aseptic conditions and the surface disinfection was made by exposure to germicidal UV light for 30 minutes [19].

Information regarding BG and BGAg characterization and composition are available at https://doi.org/10.1111/ijag.12285. Briefly, the samples were characterized by scanning electron (SEM), atomic force (AFM) microscopy, X-ray diffraction (XRD), Fourier-transform Infrared (FTIR), and surface-enhanced Raman (Raman-SERS) spectroscopy. SEM and AFM images showed particles with irregular morphology and rough surface. XRD and FTIR analyses confirmed amorphous structure corresponding to BG formation, incipient crystallization, and the presence of Si-O-Si groups typical from glass structure even with silver inclusion within BG.

### 2.2. Samples

Materials and Methods section was structured following the minimal information about T cell assay [20] and this study was approved by local ethics committee. Initial blood samples were kindly provided by three male healthy volunteers following the inclusion criteria: seronegative for HIV and HCV, vaccinated against HBV and with no signs or symptoms of acute infections at the time of blood sampling and leukocytes isolation. To ensure the safety of blood donors and maintenance of cell integrity, the specimen collection followed the guidelines established by the Clinical and Laboratory Standards Institute [21]. The healthy volunteers signed a written consent to participate according to the Helsinki Declaration of ethical guidelines.

### 2.3. Peripheral Blood Mononuclear Cells (PBMC) and Polymorphonuclear Neutrophil (PMN) Isolation and Stimulation

For PBMC and PMN isolation, 18 ml of heparinized whole blood was collected by venipuncture and aliquots of 12 ml and 9 ml were processed by density gradient centrifugation. Two different ficoll densities were applied: Histopaque® 1077, for PBMC separation, and Histopaque® 1119 for PMN isolation (Sigma-Aldrich, St. Louis, USA) [22]. The buffy coats of PBMCs and PMNs were collected and washed three times with phosphate buffer and counted in Countess® FL Automated Cell Counter (Thermo Fisher Scientific, Waltham, USA) using Trypan blue (Sigma-Aldrich, St. Louis, USA) exclusion method. Cell suspensions (PBMC and PMNs) presented at least 95% cell viability and purity as determined by morphological examination of Giemsa-stained cytocentrifuged slides (Shandon, Pittsburgh, PA, USA). Cells were suspended in equal aliquots of 2x10^6^ PBMC/ml and 10^6^ PMN/ml in RPMI 1640 medium (Gibco, Life Technologies, UK) supplemented with 10% heat-inactivated fetal bovine serum, 1% PenStrep, and 20mM HEPES. All procedures were conducted at room temperature.

### 2.4. PBMCs Viability Assay

100 *μ*l of PBMC's suspension was cultured in 96 black polystyrene wells flat bottom microplates (Greiner Bio- One, USA) and stimulated with 5 *μ*g/ml of phytohemagglutinin (PHA-P; Sigma-Aldrich, St. Louis, USA) and incubated 1:1 with BG (range 1-0.0075% wt/vl) or BGAg (range 1-0.0002% wt/vl) in culture medium for 24 hours at 37°C in a humidified atmosphere at 5% CO_2_.

Cell viability was measured using alamarBlue® according to kit protocol (Bio-Rad, Hercules, EUA). Fluorescence was measured at GloMax®-Multi Microplate Reader (Promega, Madison, USA) and percentage of viability was calculated as follows:

(1) Cytotoxicity%=FI  590  of  treated  samplesFI  590  of  untreated  cells100

where FI 590 = fluorescent intensity at 590 nm emission (560 nm excitation).

The lethal concentration 50 (LC50) was determined by semilog graph plotted as percent of untreated control for each BG and BGAg suspensions.

### 2.5. PMNs Viability Assay

Cell death was assayed by the LIVE/DEAD™ viability/cytotoxicity kit (Thermo Fisher, Rockford, IL, USA) according to kit instructions. Briefly, 10^5^ PMNs were incubated with BG and BGAg samples for 4 hours at 37°C in a humidified atmosphere at 5% CO_2_. Cells were incubated with 80% methanol for death control. Twenty minutes after staining with 1 *μ*M calcein and ethidium homodimer, fluorescence visualization was performed using epifluorescence microscope EVOS FL cell imaging system (Life Technologies, Eugene, OR, USA) equipped with a 40x objective, GFP and RFP filter cubes. Quantification of live and dead cells was analyzed in 3 aleatory fields using ImageJ (National Institutes of Health, Bethesda, MD) software according to recommendations [23].

### 2.6. Luminol-Enhanced Chemiluminescence Assay

Production of intra- and extracellular ROS was analyzed by luminol-enhanced chemiluminescence. Briefly, the PMN suspension (2x10^5^ cells/ml) was incubated for 45 min at 37°C and 5% CO_2_ with the BG and BGAg samples in white polystyrene 96-wells flat bottom (Greiner Bio-One, USA). Serum-opsonized zymosan (final concentration of 1,62 mg/ml; Sigma-Aldrich, St. Louis, USA) or medium alone were the positive and negative control, respectively. After incubation, 10^−4^ M luminol (Sigma-Aldrich, St. Louis, USA) was added and chemiluminescence was measured at 2-minute intervals with a luminometer GloMax®-Multi Microplate Reader (Promega, Madison, USA) for a period of 1h at 37°C. Chemiluminescence was expressed as relative light units (RLU) and the area under the curve (AUC) was determined for each stimulus.

### 2.7. Quantification of Cytokine Release

PBMCs (10^6^ cells/ml) were cultured for 24 hours at 48-well plates with the larger subtoxic concentration (0.031% for BG and 0.0008% for BGAg) at 37°C in 5% CO_2_. In order to induce the maximum PBMC activation and release of largest mediators amounts, PHA was used as* in vitro* model of immune cells stimulation [24]. Then, the supernatants of PBMCs cultures (with or without 5 *μ*g/ml PHA stimulation) were analyzed for IL-1*β*, TNF-*α*, IL-4, IL-6, and IL-10 concentrations by sandwich ELISA assay using OptEIA Kit (Becton Dickinson, Franklin Lakes, New Jersey, USA) according to kit protocol.

### 2.8. Statistical Analysis

Significant differences on cell viability, cytokine production, and ROS release between the groups were determined by Kruskal Wallis test with Dunn's post hoc (*α*=0.05) using the software GraphPad Prism 7 (GraphPad Software Inc., San Diego, USA).

## 3. Results and Discussion

### 3.1. Cell Viability in the Presence of BG and BGAg

**Figure 1:**
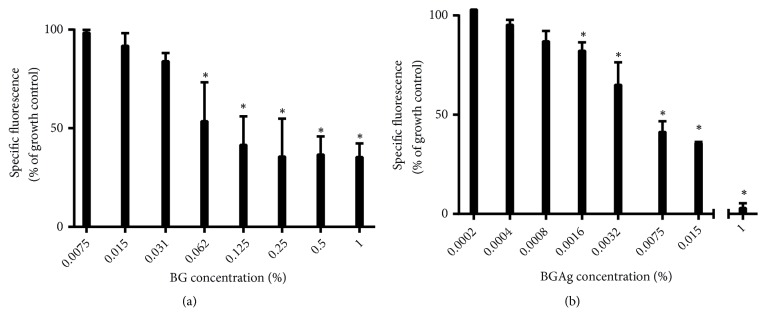
*BG (a) and BGAg (b) effects on PBMC viability*. Cell viability after treatment with increasing concentrations of BG and BGAg expressed as percentage of baseline viability. Results are shown as median with 95% confidence interval (CI) of an experiment performed in triplicate of each volunteer (^*∗*^P < 0.05 compared to growth control).

With the objective of observing acute cytotoxicity, cell viability of PBMC was accessed after 24h incubation with BG and BGAg by determining the metabolic capacity of cells to reduce the indicator dye resazurin to fluorescent resorufin. A dose-dependent reduction in cell viability was observed in both samples of BG (Figure 1). The cell viability decreased to values less than 50% of control cells at the highest treatment concentration of 0.125 and 0.0075% for BG and BGAg, respectively. Calculated LC50 values were 0.106% for BG and 0.005% for BGAg.

Over the range of BG concentrations, BG 0.031% was the highest value that did not compromise PBMC viability in comparison to growth control (P>0.05). This result is above the subtoxic concentration of 0.01% observed in a previous work [16]. The range of BGAg concentrations 1–0,0016% had a drastic effect on PBMC viability. Notably, the BGAg nontoxic concentration was 0.0008% (P>0.05) when compared with the control cells.

**Figure 2:**
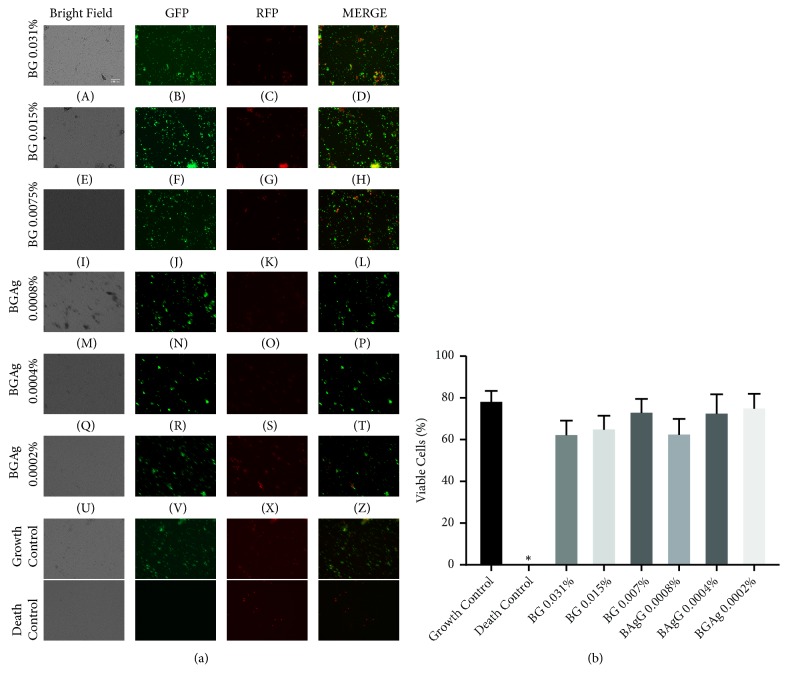
*PMN viability in presence of BG (A-L) and BGAg (M-Z)*. Demonstrative images of Live/Dead™ assay captured by 40x objective (a). The bright field shows cell morphology, GFP shows live cells green stained by calcein, and RFP shows dead cells stained by ethidium homodimer. For growth and death controls, cells were incubated, respectively, with, medium alone or 80% methanol. (b) Percentage of viable PMNs were counted on three aleatory fields. PMNs incubated with different concentrations of BG and BGAg have a similar number of viable cells compared to growth control. Results were expressed as median with 95% CI (^*∗*^P < 0.05 compared to growth control).

Therefore, these remarkable differences in cytotoxicity of the BG and BGAg against PBMCs might be associated with free Ag^2+^ in culture medium. An earlier study demonstrated that Ag^2+^ cytotoxicity against PBMCs was dose- and time-dependent [15]. BG and BGAg subtoxic doses in PBMCs did not influence the PMN viability according to LIVE/DEAD. Fluorescence images of PMN cultures stained with ethidium homodimer (damaged cell marker) did not show quantitative differences between sample wells and growth control wells (Figure 2). For avoiding PMN death due the natural short-lived cell cycle, the cell viability was quantified after incubation of 4 hours.

The discrepant results in PBMC viability may be explained by silver addiction at BG synthesis and its release in culture medium. The ion in question can induce inflammation, cell activation and oxidative stress, ROS production, protein inactivation, inhibition of respiratory chain dehydrogenase, alteration of ionic channels, misbalance of cations/anions metabolism, organelle, and DNA damage [17, 25, 26]. The soluble Ag2+ can form complexes with biomolecules causing protein dysfunction and loss of enzyme activity (inactivation, loss of tertiary structure, replacement of cofactors, exchange of structural metals, breakage of disulfide bonds, among others), impaired membrane function caused by the loss of membrane potential, mechanical damage, and interference with nutrient uptake [25]. Taken together, these events lead to cell wall breakdown and cytolysis [26]. Further probes aiming to evaluate cellular growth inhibition and quantifies cell populations as healthy, dead, apoptotic, or necrotic when exposed to BG and BGAg under different conditions of time and concentrations are necessary to complete enlighten the cytotoxic mechanism of modified bioactive glasses.

Despite undesirable effects to human cells, silver-doped glasses produced under sol–gel method were bactericidal to* Staphylococcus aureus* and* E. coli* but not toxic to human osteoblasts, under controlled concentrations [14]. Other studies [27] demonstrated growth inhibition of* S. aureus*,* E. coli*, and* P. aeruginosa* cultures under Ag^2+^ released in medium by silver-doped bioactive glasses. The antibacterial mechanism of the silver-doped bioactive glass was investigated before* E. coli* and* S. aureus* strains had DNA damage and protein denaturation compromising cellular growth [28].

Opportunistic Gram-positive staphylococci are appointed as cause of approximately 75% of osteomyelitis cases, while the most severe infections are caused by* Staphylococcus aureus *[29]. The repair of such infected bone defects is a concern in implantology and orthopedics areas. To avoid such complication and offer more predictable treatment outcomes, the association between antibacterial and osteoinductive properties is encouraged. Local delivery of alternative antimicrobials has advantages over to systemic antibiotics: broader bactericidal spectrum and nearly no resistance [30, 31]. Prevention methods as coating on implants and antimicrobial materials application are key to prevent osteomyelitis [29].

In addition, Pires et al. [18] observed that the present BGAg samples, instead of neat BG, exhibited therapeutic potential to treat infections caused by* Leishmania* parasites. The growth and proliferation inhibition of promastigote and metacyclic infective forms of the parasites occurred in the presence of 0,003% BGAg. In parallel to that study, the BGAg effective concentration allowed a PBMC viability of 65.5% after 24 hours of incubation. However, for other cell types, e.g., osteoblasts and fibroblasts, this relationship between therapeutic concentrations and cell viability lacks definition.

### 3.2. Oxidative Stress

**Figure 3:**
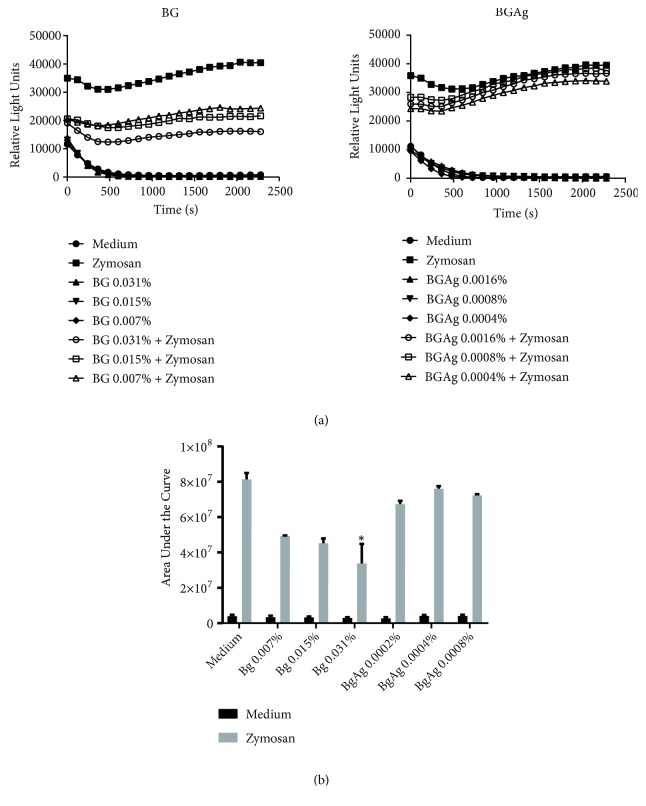
*ROS production in PMNs*. (a) Chemiluminescent curves showing similar relative light units (RLU) detection between BG and BGAg samples when compared to with negative control (medium only). BG samples decreased ROS production when coincubated with zymosan (positive control). Each point represents the median of triplicate readings of ex vivo PMN cultures (n=3). (b) Area under the curve (AUC) values plotted for each stimulus. The sample BG 0.031% reduced significantly ROS production in comparison with only zymosan as stimulus. Values were expressed as median with CI (^*∗*^P < 0.05 compared to positive control).

Both samples of BG and BGAg alone were unable to induce intra- and extracellular ROS production above baseline parameters (Figure 3). Therefore, the higher dilution of neat BG decreased ROS detection when coincubated with opsonized zymosan. This compound activates an oxidative burst by binding itself to complement receptors, leading transduction signal to protein kinase C activation and consequent activation of NADPH-oxidase, the key enzyme of oxidative burst [32]. The oxidative stress reduction could be explained by the following: BG dissolved products like silica, calcium, phosphate, and sodium ions contribute to the balance of the oxidative status, or they may interfere with zymosan-receptor complex, or they might have the ability to act as free radicals and superoxides scavengers. The* in vivo* redox activity of bioglass compounds was previously reported [30]; thus the exact mechanism of action is still not clear. Such modulatory effect is of great relevance in osteogenesis by induction of osteoblasts metabolism and differentiation.

On the other hand, ROS production is a common finding on* in vitro* and* in vivo* models due to Ag presence in different biological systems. Overproduction of free radicals is appointed as a mechanism of cytotoxicity by oxidative stress, resulting in genotoxicity and cells breakdown [25]. BGAg samples unhanged ROS levels in culture medium; this finding may justify why the concentrations applied were not cytotoxic for PMN's cultures.

### 3.3. Cytokine Modulation

**Figure 4:**
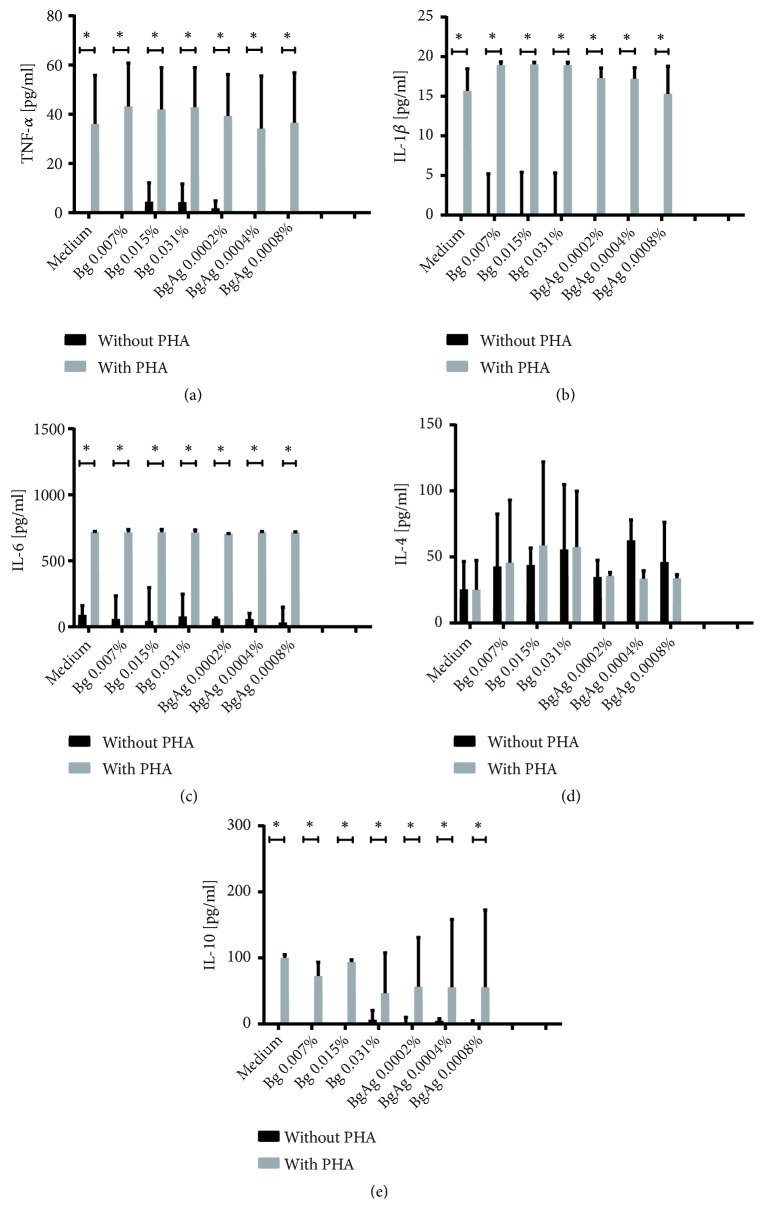
*Cytokine release by PBMC cultures incubated with BG or BGAg*. Titration of TNF-*α* (a), IL-1*β* (b), IL-6 (c), IL-4 (d), and IL-10 (e) released at 24-hour culture supernatant of PBMC's cultured with or without 5 *μ*g/ml PHA stimulation. There were no significant differences on cytokine's production between BG and BGAg stimulus and medium alone treatment. Bioactive glasses were unable to reduce cytokine levels after PHA coincubation.  ^*∗*^Correspond to the statistical difference (p<0,05). Data were presented as the median ± 95% CI of triplicates of each volunteer (n=3).

Quantification of TNF-*α*, IL-1*β*, IL-6, and IL-10 at 24h PBMC's culture supernatant performed by sandwich ELISA showed no significant differences between the treatments with three subtoxic BG and BGAg suspensions and baseline control (Figure 4). At some sample concentrations, TNF-*α*, IL-1*β*, and IL-10 release were lower than detection limits of the method. Interestingly, however, all BG and BGAg samples induced IL-4 production to similar levels than PHA stimulus (Figure 4). This result cannot be attributed to the action of biomaterials since the production of IL-4 by unstimulated cells was not significantly different. The presence of bioactive glasses did not change TNF-*α*, IL-1*β*, IL-6, and IL-10 secretion profile compared to basal levels or PHA stimulated cells. These results suggest that bioactive glasses particles even when doped with silver ions do not change the levels of releasing proinflammatory and anti-inflammatory cytokines by human PBMC.

Immunomodulatory effects of bioactive glasses were investigated by previous studies. Particles belonging to system 60S did not change significantly IL-4 secretion profile by PBMCs [33]. In agreement with our current results, other studies found that 45S5 glass did not interfere with IL-6, IL-10, and TNF-*α* secretion by nonstimulated macrophages and monocytes cultures [7]. This same study observed a decrease in TNF-*α* production when the cells were incubated with LPS. On the other hand, another study showed that 45S5 powders upregulated TNF-*α* secretion by peritoneal macrophages [34]. Beyond cell population variances, differences on cytokine modulation may be explained by different factors that induce immune response by biomaterials, such as BG composition, particle size, surface chemistry, plasma protein binding, and exposure model [35].

The literature has a great extended relates about therapeutic perspectives for bioactive glasses, including implant coatings, alloplastic grafts for sinus lift (micro particles formulation) or replacement after tumor removal (scaffolds), and dental composites [3]. Beyond the hard tissues engineering, bioactive glasses can also be applied in the soft tissue manipulation. Several studies report on the application of BGs for wound healing by mechanisms of stimulation of angiogenesis, establishment of bg-collagenous bonding, and accelerated rate of blood coagulation [36]. The described biological properties are relevant in the context of management of chronic wounds including, for example, diabetic foot ulcers, venous leg ulcers, and pressure ulcers [37].

In some therapies against cancer, arthritis, and allergies, an immunomodulatory capacity of the therapeutic agent is highly desirable. However, an unbalanced immunosuppression or immunostimulation might be associated with many of the undesirable side effects observed in most cases. Thus, the study of interactions between biomaterials and the immune system is key for safe medicinal use of recently developed biomaterials. A recent work questioned the actual capacity to examine the real function of biomaterials within both innate and adaptive immune responses, mainly concerning the B and T cell responses [38]. Although models for determining acute and long-term immune toxicities have been developed, studies on the treatment and prediction of immunomodulatory activity are scarce [35, 38]. One study showed that some biomaterials modified the adaptive immunity (cell phenotype and cytokine release) and promoted tissue repair [39]. Our results follow these studies which contribute to expanding the knowledge about materials science and biomedical engineering applications in humans [40].

## 4. Conclusions

The presence of silver increased the glass cytotoxicity against human PBMCs. The 58S BG and BGAg subtoxic concentrations did not interfere with patterns associated with release of main regulatory, pro- and anti-inflammatory cytokines by cultured PMBCs. Both BG and BGAg were unable to induce ROS production, while neat BG decreased ROS production when coincubated with serum-opsonized zymosan, suggesting its potential scavenger activity. Further studies of silver dissolution in culture medium,* in vivo* Ag^+^ biodistribution and the development of mechanisms for ion release control according to desirable dose are necessary and important next steps to increase our current knowledge about therapeutic applications of BG and BGAg.

## Data Availability

Previously reported BG and BGAg synthesis and characterization data were used to support this study and are available at https://doi.org/10.1111/ijag.12285. This prior study is cited at relevant places within the text as [18].
